# Increased phosphorylation of collapsin response mediator protein-2 at Thr514 correlates with β-amyloid burden and synaptic deficits in Lewy body dementias

**DOI:** 10.1186/s13041-016-0264-9

**Published:** 2016-09-08

**Authors:** Huayang Xing, Yun-An Lim, Joyce R. Chong, Jasinda H. Lee, Dag Aarsland, Clive G. Ballard, Paul T. Francis, Christopher P. Chen, Mitchell K. P. Lai

**Affiliations:** 1 Department of Pharmacology, Yong Loo Lin School of Medicine, National University of Singapore, Unit 09-01, Centre for Translational Medicine (MD6), 14 Medical Drive, Kent Ridge, 117599 Singapore; 2 Memory, Ageing and Cognition Centre, National University Health System, Kent Ridge, Singapore; 3 Department of Neurobiology, Care Sciences and Society, Alzheimer’s Disease Research Centre, Karolinska Institutet, Novum, Stockholm, Sweden; 4 Center for Age-Related Diseases, Stavanger University Hospital, Stavanger, Norway; 5 King’s College London, Wolfson Centre for Age-Related Diseases, London, UK

**Keywords:** Collapsin response mediator protein-2, Dementia with Lewy bodies, Parkinson’s disease dementia, Axonal pathology

## Abstract

Collapsin response mediator protein-2 (CRMP2) regulates axonal growth cone extension, and increased CRMP2 phosphorylation may lead to axonal degeneration. Axonal and synaptic pathology is an important feature of Lewy body dementias (LBD), but the state of CRMP2 phosphorylation (pCRMP2) as well as its correlations with markers of neurodegeneration have not been studied in these dementias. Hence, we measured CRMP2 phosphorylation at Thr509, Thr514 and Ser522, as well as markers of β-amyloid (Aβ), tau-phosphorylation, α-synuclein and synaptic function in the postmortem neocortex of a longitudinally assessed cohort of LBD patients characterized by low (Parkinson’s disease dementia, PDD) and high (dementia with Lewy bodies, DLB) burden of Alzheimer type pathology. We found specific increases of pCRMP2 at Thr514 in DLB, but not PDD. The increased CRMP2 phosphorylation correlated with fibrillogenic Aβ as well as with losses of markers for axon regeneration (β-III-tubulin) and synaptic integrity (synaptophysin) in LBD. In contrast, pCRMP2 alterations did not correlate with tau-phosphorylation or α-synuclein, and also appear unrelated to immunoreactivities of putative upstream kinases glycogen synthase kinase 3β and cyclin-dependent kinase 5, as well as to protein phosphatase 2A. In conclusion, increased pCRMP2 may underlie the axonal pathology of DLB, and may be a novel therapeutic target. However, antecedent signaling events as well as the nature of pCRMP2 association with Aβ and other neuropathologic markers require further study.

**Electronic supplementary material:**

The online version of this article (doi:10.1186/s13041-016-0264-9) contains supplementary material, which is available to authorized users.

## Introduction

Collapsin response mediator protein-2 (CRMP2) is the first identified member of the collapsin response mediator protein (CRMP) family [1]. There are five known isoforms, CRMP1-5, of which only CRMP2 (also known as dihydropyrimidinase-like 2) expression remains high in the adult brain [2, 3] and is known to promote axonal pathfinding during neural development by mediating semaphorin 3A-induced growth cone collapse [1]. Physiologically, CRMP2 is also involved in neurotransmitter release, neuronal migration, as well as in axonal transport and guidance [4–7]. CRMP2 can be phosphorylated at Ser522 by cyclin-dependent kinase 5 (CDK5), which in turn facilitates glycogen synthase kinase 3β (GSK3β)-mediated phosphorylation at Thr509 and Thr514 [7–10]. CDK5 can be activated by p35 or its calpain cleavage product, p25 [11], while GSK3β activity is activated by phosphorylation at Tyr216 and inhibited by phosphorylation at Ser9 [12]. Phosphorylation of CRMP2 decreases CRMP2 binding activity to tubulin, thereby inhibiting neurite outgrowth [10] and potentially leading to axonal degeneration [13], whilst dephosphorylation of CRMP2 by protein phosphatase 2A (PP2A) enhances axonal growth [14]. Axonopathy and associated synaptic dysfunction are early features of Alzheimer’s disease (AD), currently the commonest cause of neurodegenerative dementia in the elderly [15]. Besides synaptic deficits, the neuropathological hallmarks of AD include aggregated β-amyloid (Aβ)-containing senile plaques and neurofibrillary tangles consisting of hyperphosphorylated tau proteins. Interestingly, CRMP2 phosphorylation has been found to be increased in AD, suggesting that CRMP2 phosphorylation is associated with Aβ pathology, and that CRMP2 dysregulation may be one mechanism underlying axonopathy and neurite degeneration [16, 17]. Besides AD, Lewy body dementias (LBD) constitute the second largest cause of neurodegenerative dementias [18] and encompass both Parkinson’s disease with dementia (PDD) and dementia with Lewy bodies (DLB). PDD and DLB share similar neuropathological features, especially with respect to presence of cortical aggregated α-synuclein-containing Lewy bodies [19], and differentiation is mainly clinical, based on the “one-year rule” where patients presenting with dementia before, or within a year of, parkinsonism are diagnosed with DLB, while dementia occurring > 1 year after parkinsonism is considered to be PDD [20]. Importantly however, PDD has relatively low burden of Aβ plaques, while DLB has variable but generally higher Aβ burden [21–23]. However, the status of CRMP2 in LBD, and whether CRMP2 phosphorylation is related to Aβ and /or α-synuclein load is at present unclear. As there is as yet no licensed treatment for DLB, we aimed to assess if CRMP2 phosphorylation may represent a potential therapeutic target. Therefore, we measured CRMP2 phosphorylation, Aβ, tau phosphorylation and α-synuclein, together with markers of axonal and synaptic deficits in the postmortem neocortex of a cohort of longitudinally assessed PDD and DLB patients.

## Results

### Demographic and disease variables of the study cohort

Table 1 lists the demographic and disease variables of the control, DLB and PDD, indicating that subjects were well-matched in age, postmortem delay and brain pH (mean values ranging from 6.3-6.5), with the latter used as an indicator of tissue quality, and pH above 6.1 considered acceptable [24]. In agreement with findings of higher AD pathological burden in DLB [23], higher proportions of Braak stage V/VI were found in DLB than PDD, while none of the control subjects were staged higher than Braak III (Table 1).

**Table 1:** Demographic and disease variables of control and LBD subjects

Demographics		Control	PDD	DLB
Maximum *N*		19	19	20
Gender (Male/Female)		11/8	8/11	10/10
Age at Death (years)		81.8 ± 1.5	80.1 ± 1.4	81.0 ± 1.3
Postmortem Interval (hours)^a^		38.6 ± 5.6	31.4 ± 3.8	35.0 ± 5.8
Brain pH		6.46 ± 0.06	6.44 ± 0.06	6.37 ± 0.06
Disease variables				
Braak Stage^b^	0	3	1	0
	I/II	11	13	4
	III/IV	1	3	8
	V/VI	0	2	8

Data are expressed as mean ± SEM. *NA* not available

^a^Postmortem interval data was not available for one PDD patient

^b^Four controls were not Braak staged

### CRMP2 is enriched in soluble cytosolic fractions of postmortem human neocortex

CRMP2 is known to be abundantly localized to the cytosol as a cytoskeletal associated protein [25], which has previously been shown to be enriched in cytosolic soluble fractions, along with actin [26, 27]. To further characterize CRMP2 distribution, equal amounts of protein from total and cytosolic fractions of brain homogenates were immunoblotted for CRMP2. As expected, CRMP2 immunoreactivity was enriched in the cytosolic fraction, along with β-actin (Additional file 1: Figure S1). We therefore measured total and phosphorylated CRMP2 in the cytosolic, soluble fractions for correlations with soluble Aβ peptides from the same fractions (see below).

### Specific increases of CRMP2 phosphorylation at Thr514 in DLB

**Fig. 1:**
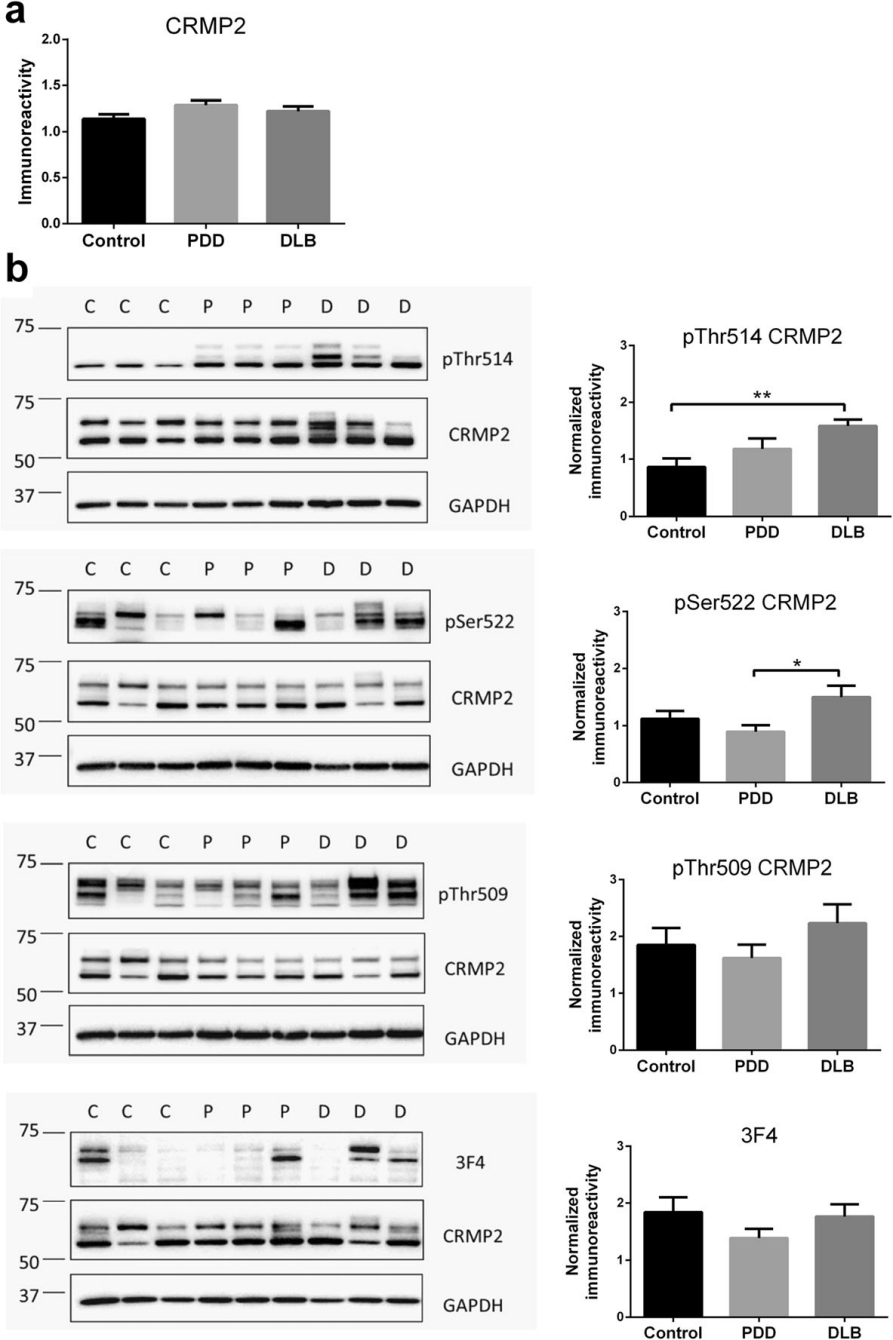
Increased CRMP2 phosphorylation at Thr514 in LBD parietal cortex. **a** Bar graph of total CRMP2 immunoreactivity (mean ± SEM in arbitrary units). **b** Representative immunoblots (with molecular weight indicators in kDa to the left of the immunoblots) and bar graphs of pCRMP2 immunoreactivities (mean ± SEM in arbitrary units) at the indicated epitopes, with GAPDH as loading control. Available *N* for control (C) = 19; PDD (P) = 19 and DLB (D) = 20. *p < 0.05; **p < 0.01, significant differences for multiple pair-wise comparisons (one-way ANOVA with Bonferroni *post*-*hoc* tests)

Because previous studies on AD have reported increased CRMP2 phosphorylation which may be related to amyloid burden [16, 17], we were interested in studying these associations in LBD, and compared the immunoreactivities of cytosolic phosphorylated CRMP2 between the two clinical subgroups known to manifest relatively high (DLB) and low (PDD) cortical Aβ [21–23]. Interestingly, while total CRMP2 levels were unchanged (Fig. 1a), pThr514 CRMP2 was increased in DLB (Fig. 1b). In contrast, phosphorylated CRMP2 at Thr509, Ser522, or triply phosphorylated Thr509, Ser518 and Ser522 as recognized by the 3 F4 antibody [7] were not significantly altered in either DLB or PDD compared to controls (Fig. 1b). pThr514 CRMP2 was similarly increased in DLB, but not PDD in the total homogenate fractions (Additional file 2: Figure S2).

### Increased pThr514 CRMP2 correlates with Aβ_42_ to Aβ_40_ ratio in DLB

**Fig. 2:**
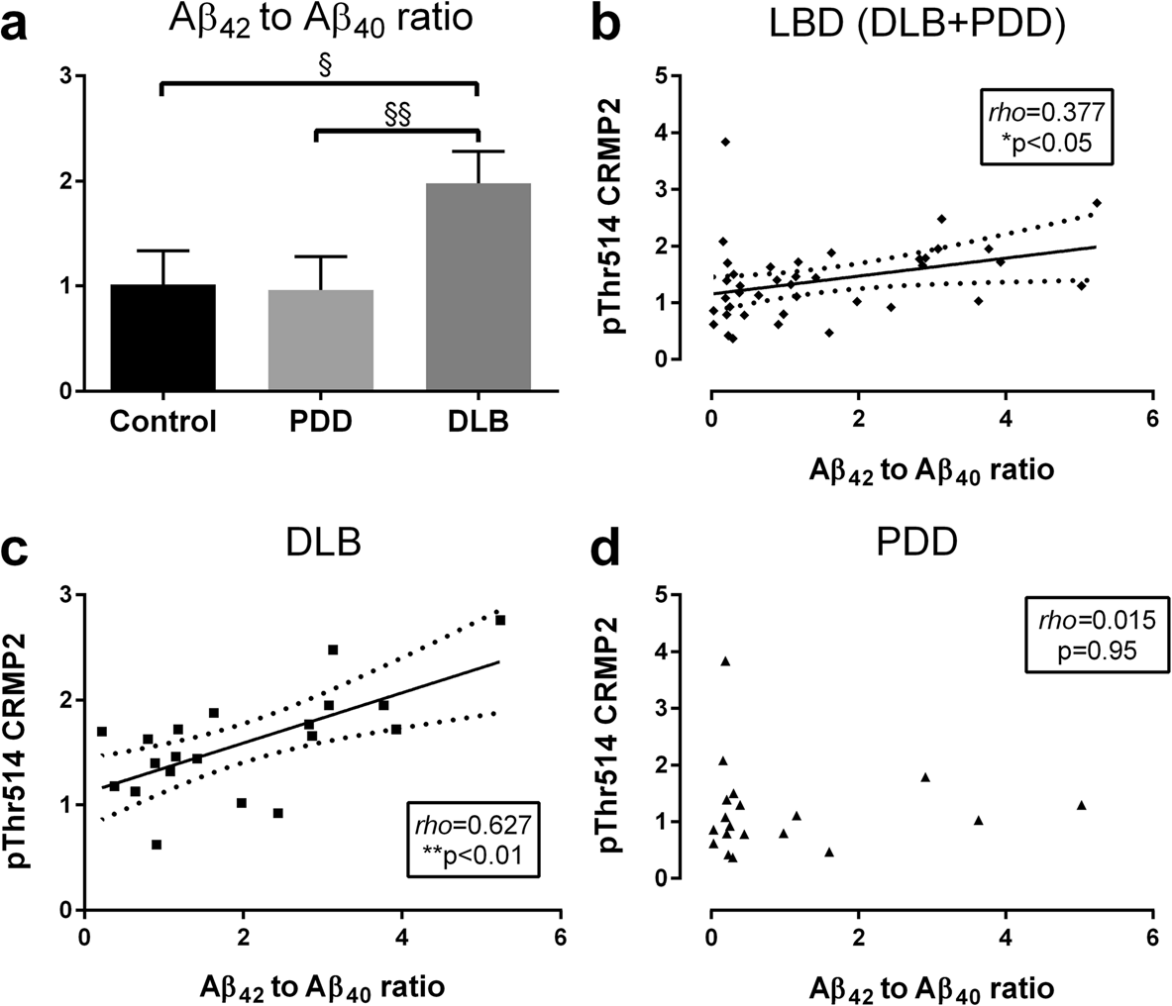
Increased pThr514 CRMP2 correlates with Aβ_42_ : Aβ_40_ in LBD parietal cortex. **a** Bar graph of mean (± SEM) Aβ_42_ to Aβ_40_ ratios. Scatter plots of pThr514 CRMP2 with Aβ_42_ : Aβ_40_ in cytosolic fractions of **b** LBD (DLB + PDD), **c** DLB and **d** PDD parietal cortex, with insets indicating *rho* and *p* values. Where a correlation is significant, a best-fit regression line (solid line) is added together with its 95 % prediction intervals (*dotted lines*). Available *N* for control = 19; PDD = 19 and DLB = 20. ^§^ *p* < 0.05; ^§§^ *p* < 0.01, significant differences for multiple pair-wise comparisons (Kruskal-Wallis H with Dunn’s *post*-*hoc* tests). *p < 0.05; **p < 0.01, significant Spearman correlations

The 42 amino-acid species of Aβ (Aβ_42_) is more fibrillogenic and prone to forming toxic oligomers compared to Aβ_40_ [28], and is the major component of amyloid plaques, a pathological hallmark of AD. The Aβ_42_ to Aβ_40_ ratio (Aβ_42_ : Aβ_40_) is therefore used as an indicator of neurotoxicity and disease severity in AD [29]. Aβ_42_ : Aβ_40_ is also a biomarker for AD clinical assessment and has been shown to be more consistently associated with cognitive impairments than only Aβ_42_ or Aβ_40_ [30, 31]. Corroborating previous studies on the differences in amyloid burden between DLB and PDD [21–23], we found that Aβ_42_ : Aβ_40_ in BA40 was significantly increased in DLB, but not PDD (Fig. 2a). The Aβ_42_ : Aβ_40_ correlated with pThr514 CRMP2 immunoreactivity in the combined LBD (DLB + PDD) group (Fig. 2b), an observation which seemed to be driven predominantly by DLB (Fig. 2c) and not PDD (Fig. 2d). In contrast, none of the other pCRMP2 species measured were correlated with Aβ_42_ : Aβ_40_ (Additional file 3: Figure S3). Additionally, measures of tau phosphorylated at Ser396 (a marker for neurofibrillary tangle burden [32]), α-synuclein monomers, as well as the insoluble, potentially pathogenic pSer129 α-synuclein [33] did not correlate with pThr514 CRMP2 (Additional file 4: Figure S4, Additional file 5: Figure S5 and Additional file 6: Figure S6) or with other pCRMP2 species (data not shown).

### Increased pThr514 CRMP2 correlates with synaptic deficits in LBD

**Fig. 3:**
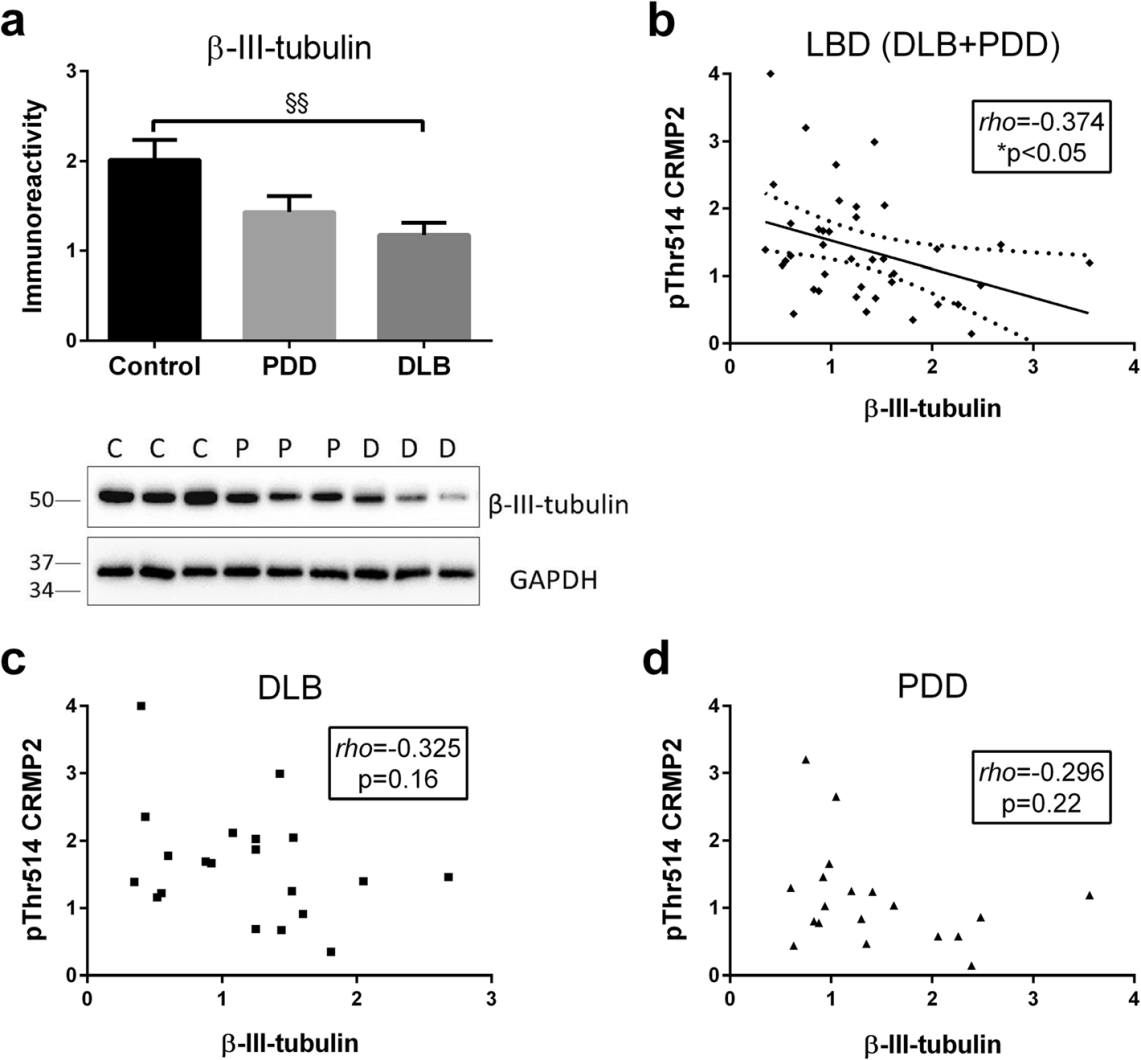
Increased pThr514 CRMP2 correlates with β-III-tubulin loss in in LBD parietal cortex. **a** Bar graph of β-III-tubulin immunoreactivity (mean ± SEM in arbitrary units) and representative immunoblots, with GAPDH as loading control. Scatter plots of pThr514 CRMP2 with β-III-tubulin immunoreactivities in total homogenate fractions of **b** LBD (DLB + PDD), **c** DLB and **d** PDD parietal cortex, with insets indicating *rho* and *p* values. Where a correlation is significant, a best-fit regression line (solid line) is added together with its 95 % prediction intervals (dotted lines). Available *N* for control (C) = 19; PDD (P) = 19 and DLB (D) = 20. ^§§^ *p* < 0.01, significant difference for multiple pair-wise comparisons (one-way ANOVA with Bonferroni *post*-*hoc* tests). *p < 0.05, significant Spearman correlation

**Fig. 4:**
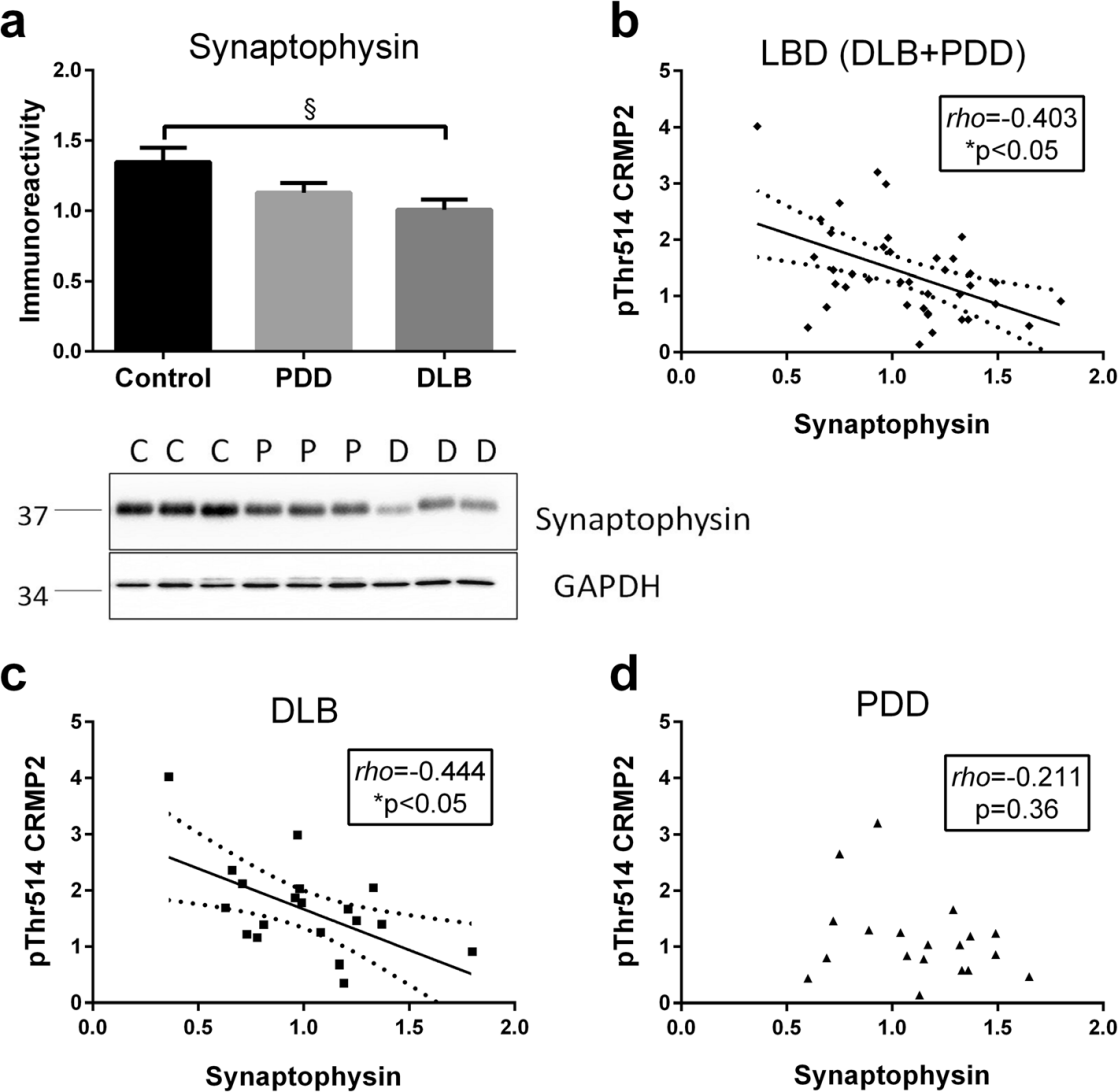
Increased pThr514 CRMP2 correlates with synaptophysin loss in in LBD parietal cortex. **a** Bar graph of synaptophysin immunoreactivity (mean ± SEM in arbitrary units) and representative immunoblots, with GAPDH as loading control. Scatter plots of pThr514 CRMP2 with synaptophysin immunoreactivities in total homogenate fractions of **b** LBD (DLB + PDD), **c** DLB and **d** PDD parietal cortex, with insets indicating *rho* and *p* values. Where a correlation is significant, a best-fit regression line (solid line) is added together with its 95 % prediction intervals (dotted lines). Available *N* for control (C) = 19; PDD (P) = 19 and DLB (D) = 20. ^§^ *p* < 0.05, significant difference for multiple pair-wise comparisons (one-way ANOVA with Bonferroni *post*-*hoc* tests). *p < 0.05, significant Spearman correlations

CRMP2 phosphorylation, including pThr514, is known to deactivate CRMP2 and affect axonal growth cone as well as associated synaptic function and plasticity [10, 34, 35]. To study possible synaptic effects of increased pThr514 CRMP2, we correlated pThr514 immunoreactivity with markers of regenerating axon/growth cone (β-III-tubulin [36, 37]) and synaptic integrity (synaptophysin [38]). Both β-III-tubulin and synaptophysin were decreased in DLB, with intermediate values in PDD (Figs. 3a and 4a). These results indicate the presence of synaptic deficits in LBD, especially with concomitant Aβ burden. Interestingly, pThr514 CRMP2 negatively correlated with both β-III-tubulin and synaptophysin in LBD (Figs. 3b and 4b), although for β-III-tubulin the correlations were not significant within the dementia subgroups (Fig. 3c and d), while correlations remained significant in DLB, but not PDD, in the case of synaptophysin (Fig. 4c and d). Taken together, the data suggest that increased pThr514 CRMP2 is associated with deficits in axonal regeneration and synaptic integrity in LBD.

### Alterations of CDK5 activator p25 and GSK3β in LBD

**Fig. 5:**
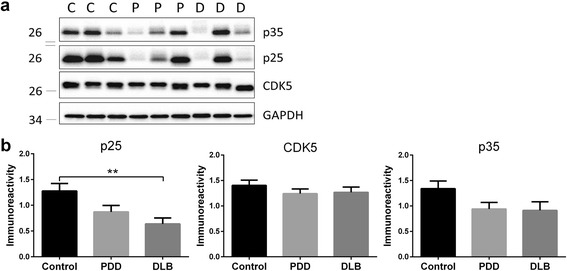
Decreased CDK5 activator, p25 in DLB parietal cortex. **a** Representative immunoblots and **b** bar graphs of p25, p35 and total CDK5 immunoreactivities (mean ± SEM in arbitrary units), with GAPDH used as a loading control. Available *N* for control (C) = 19; PDD (P) = 19 and DLB (D) = 20. **p < 0.01, significant difference for multiple pair-wise comparisons (one-way ANOVA with Bonferroni *post*-*hoc* tests)

**Fig. 6:**
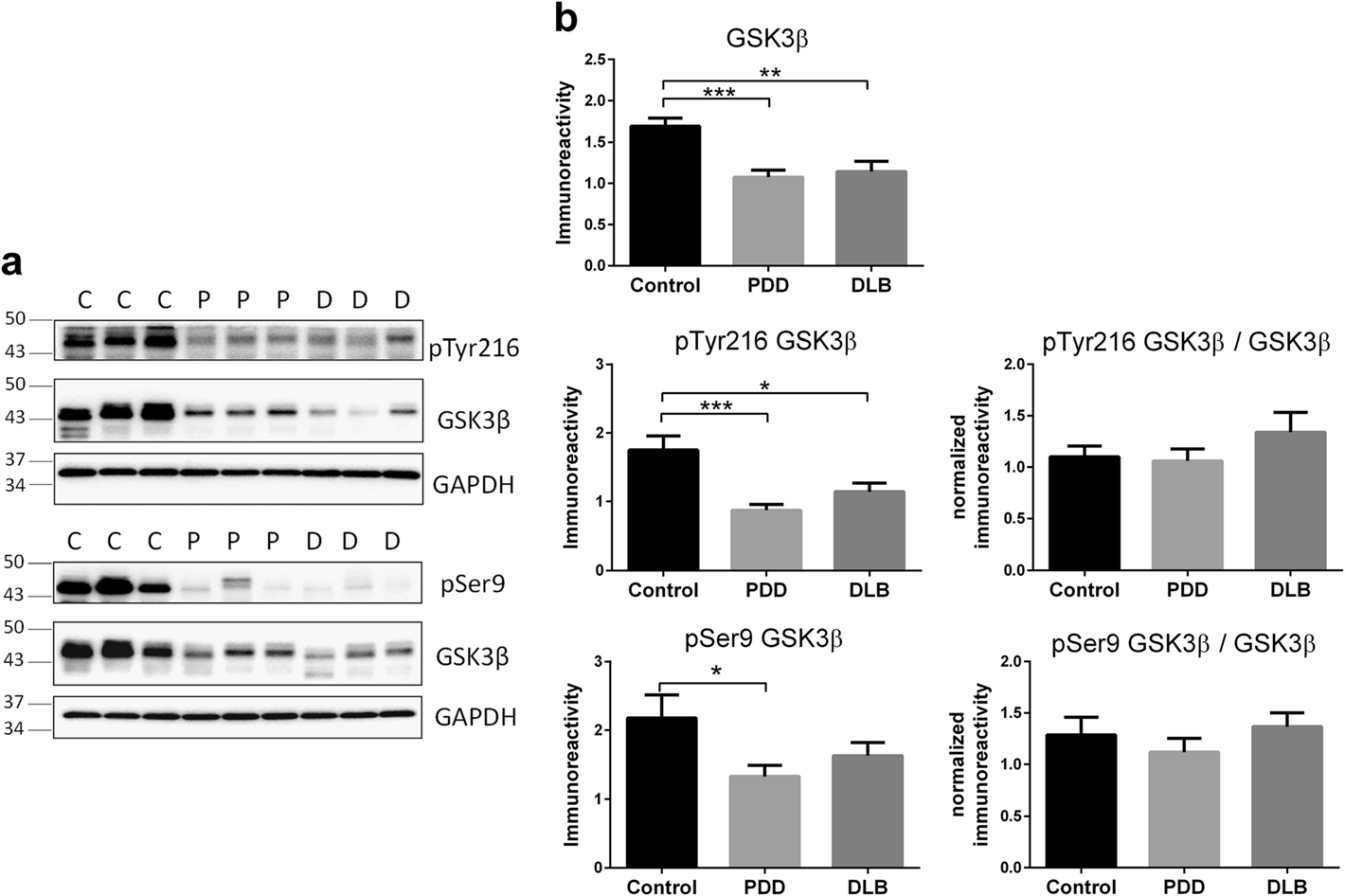
Decreased GSK3β immunoreactivities in LBD parietal cortex. **a** Representative immunoblots and **b** bar graphs of immunoreactivities (mean ± SEM in arbitrary units) of total and phosphorylated GSK3β (pTyr216 and pSer9), as well as immunoreactivities of phosphorylated GSK3β normalized to total GSK3β, with GAPDH used as loading control. Available *N* for control (C) = 19; PDD (P) = 19 and DLB (D) = 20. *p < 0.05; **p < 0.01; ***p < 0.001, significant difference for multiple pair-wise comparisons (one-way ANOVA with Bonferroni *post*-*hoc* tests)

To investigate whether the increased pThr514 CRMP2 may be associated with changes in upstream kinases, we measured total CDK5 and GSK3β as well as indicators of activation (p25, p35, pTyr216 GSK3β) and inactivation (pSer9 GSK3β). Furthermore, given that PP2A can dephosphorylate CRMP2 at Thr514 [14], the catalytic C-subunit of PP2A was also assessed. Figure 5 shows that while CDK5 levels were unchanged in LBD, there was a loss of p25 in DLB, while p35 showed similar trends not reaching statistical significance. In contrast, GSK3β levels were reduced in both PDD and DLB (Fig. 6), together with decreases of GSK3β phosphorylation at both Tyr216 (PDD and DLB) and Ser9 (PDD). However, when normalized to total GSK3β, neither pTyr216 GSK3β/GSK3β nor pSer9 GSK3β/GSK3β was significantly different between the groups (Fig. 6), indicating the loss of total GSK3β immunoreactivity rather than changes in activation status. Lastly, no alterations of PP2A C-subunit was observed in LBD (Additional file 7: Figure S7), suggesting that altered pThr514 CRMP2 may not be related to changes in dephosphorylation activity.

## Discussion

The CRMP2 member of the collapsin response mediator protein family has well-established roles in various synaptic functions, including regulation of neurotransmitter release, axonogenesis and axon guidance [4–7]. It is therefore not surprisingly that CRMP2 alterations have been well characterized in AD [16, 17, 39] where synaptic dysfunction represents a prominent pathophysiological feature [40]. However, CRMP2 alterations have not been studied in LBD. In this study, we showed in DLB parietal cortex a specific increase of CRMP2 phosphorylation at Thr514 (Fig. 1), which is known to reduce CRMP2 binding affinity to molecules involved in axonal growth and microtubule dynamics, thereby inhibiting axonal function and leading to axonal pathology [6, 41–44]. The increased pThr514 CRMP2 correlated with Aβ burden (as denoted by Aβ_42_ : Aβ_40_, see Fig. 2) as well as with reductions in markers of axonal regeneration and synaptic integrity (Figs. 3 and 4). Therefore, increased CRMP2 phosphorylation may be one mechanism underlying the detrimental effects of Aβ on synapses in LBD, similar to that seen in AD [16, 17]. There has been an ongoing debate on whether the two clinical subtypes of LBD (PDD and DLB), whilst distinguishable by the one-year rule and other cognitive features [20], are in fact different disease entities, or are different manifestations in the spectrum of LBD [19, 45–47]. In this study, we showed both increases in pThr514 CRMP2 (Fig. 1) and decreases in synaptic markers (Figs. 3 and 4) to be more severe in LBD, which is known to have relatively higher Aβ burden; while PDD, which has a low Aβ burden, showed intermediate changes not reaching statistical significance [21–23] (also see Fig. 2a). Interestingly, PDD and DLB are known to have similar α-synuclein loads as well as Lewy body burden [19, 48, 49] (also see Additional file 5: Figure S5), suggesting that at least some of the identified neurochemical differences between PDD and DLB could be driven by the different Aβ burden in the subtypes. This postulate is recently supported by the finding that low cerebrospinal fluid Aβ42 values predicted a more rapid cognitive decline in DLB [50]. The clinical implications of this postulate include the potential efficacy of novel AD pharmacotherapeutics in DLB, particularly those targeting Aβ-related disease process, and the impetus to include DLB patients alongside AD patients in trials of such therapeutics. Furthermore, suppression of CRMP2 phosphorylation has been found to improve learning and memory deficits in an AD model [51] and ameliorate axonopathy in a multiple sclerosis model [13], thus pointing to CRMP2 as a therapeutic target in axonopathy-related neurodegenerative diseases. Our study now extends the potential pathogenicity of CRMP2 phosphorylation to the axonal pathology of DLB [52, 53].

While the current study investigated a comprehensive range of CRMP2 neurochemical markers and neuropathological correlates, there are several limitations and questions which require follow-up investigations. First, due to issues of tissue availability, we limited our measurements to the parietal cortex (BA40). Although previous studies have shown similar involvement of plaques, tangles and α-synuclein pathology between BA40 and other neocortical regions like the frontal or temporal cortex [23], it is unclear whether the findings in BA40 are representative of other areas, and follow-up measurements of CRMP2 in various brain regions will be needed. Similarly, we showed that markers of neurofibrillary tangles (pSer396 tau) are increased in DLB, akin to observations in AD [54]. However, pSer396 tau changes did not reach statistical significance due to variability (see Additional file 4: Figure S4a), and higher *N*’s may be needed to have sufficient power to study potential correlations between different pCRMP2 species and tau phosphorylation, especially since certain pCRMP2, e.g., 3 F4 epitopes, are known to be associated with neurofibrillary tangles [55]. With regards to α-synuclein, we found no difference in levels of monomeric species in agreement with previous studies [48, 49], and no correlations between α-synuclein and pCRMP2 (Additional file 5: Figure S5). In contrast, α-synuclein phosphorylated at Ser129 in insoluble fractions was significantly elevated in DLB, whilst PDD showed intermediate levels, and may be more specific to [56], and pathogenic in [57], LBD, similar with previous finding [58] (Additional file 6: Figure S6). However, this insoluble pSer129 α-synuclein also did not correlate with pThr514 CRMP2 level, suggesting this pCRMP2 dysregulation may be directly associated with Aβ, rather than indirectly via pSer129 α-synuclein effects of Aβ deposition [58]. However, the observed differences in pSer129 α-synuclein in DLB versus PDD are interesting, and their potential pathogenic and therapeutic implications should be further investigated.

It would also be of interest to investigate the specificity of increased CRMP2 phosphorylation at the pThr514 epitope, although this may, again, be due partly to the need for higher *N*’s, as pCRMP2 at pSer522 and pThr509 showed trends toward increases (Fig. 1b). Furthermore, the mechanisms underlying Aβ’s effects on increased phosphorylation at the Thr514 (and possibly other) site are currently unclear, as the protein kinases known to phosphorylate CRMP2 showed alterations which suggest lower, rather than higher, enzymatic activities. For example, while CDK5 levels were unaltered, there was a significant decrease of its activator, p25 in DLB (Fig. 5b). On the other hand, GSK3β immunoreactivities were lost in both PDD and DLB, and while phosphorylated GSK3β (both activating pTyr216 and inactivating pSer9) epitopes were reduced, they seemed to reflect loss of GSK3β proteins rather than any change in activation status (Fig. 6b). It is worth noting that GSK3β loss has been reported in AD and Huntington’s disease [59, 60], and this study adds both the PDD and DLB subtypes of LBD to the list. Additionally, p25/p35 have also been reported to be decreased in AD [61, 62], although these findings are not unanimous, as unchanged p35 (together with undetectable p25) has also been reported [63]. In contrast to the current findings in LBD, reductions of GSK3β correlated with decreased pCRMP2 in Huntington’s disease [60]. Because PP2A levels were also unchanged in LBD (Additional file 6: Fig. 6b), the increased pCRMP2 in LBD does not seem to be associated with CDK5, GSK3β or PP2A changes, but may reflect the activities of other, as yet uncharacterized, kinases or phosphatases. Therefore, whether the observed reductions of GSK3β and p25 are adaptive plasticity processes in response to increased pCRMP2, or are related to other disease process like neuronal apoptosis [64], requires further study.

In conclusion, this study demonstrates an increase of CRMP2 phosphorylation in the DLB parietal cortex specifically at Thr514, which may be related to fibrillogenic Aβ load as well as to deficits in axonal growth and synaptic integrity. CRMP2 phosphorylation therefore represents a mechanism of axonal pathology in DLB with concomitant Alzheimer pathology, which has implications in the potential utility of Aβ- or CRMP2-targeting therapies in this subtype of LBD. However, further studies are needed to (1) validate the observed pCRMP2 changes in other brain regions in a larger cohort; (2) elucidate the mechanisms linking Aβ to pCRMP2 in LBD; and (3) investigate potential associations between CRMP2 function and other pathological features, including various different α-synuclein species.

## Methods

### Patients and brain tissues

All subjects for this study were selected based on clinicopathological consensus diagnoses, including the “one-year rule” [20] and the Movement Disorders Society criteria [65] to distinguish between PDD and DLB, the two major subtypes of LBD. At death, informed consent was sought from next-of-kin before removal of brains, and tissues were collected via the Thomas Willis Oxford Brain Collections, the London Neurodegenerative Diseases Brain Bank, Newcastle University and University Hospital Stavanger, the UK sites being part of the Brains for Dementia Research network. For this study, tissues from inferior parietal lobe (Brodmann area, BA40) were collected from 19 aged controls, 19 PDD and 20 DLB subjects, all of whom had been neuropathologically assessed by standardized grading instruments, including Braak staging [66] and the Newcastle/McKeith criteria for Lewy body disease [20] (see also Howlett et al. [23]). Not all clinical or neurochemical variables were available for all samples, and respective *N* values are listed in the table and figure legends.

### Tissue processing and brain homogenate preparation

All reagent grade chemicals were purchased from Sigma-Aldrich (USA) unless otherwise stated. Blocks of frozen brain tissues (around 1 cm^3^) were thawed on ice and dissected free of meninges and white matter with disposable surgical scalpels. After dissection, samples were homogenized (50 mg tissue wet weight/mL) with an Ultra-Turrax homogenizer (IKA, Germany, maximum setting, 10 s) in ice-cold homogenizing buffer (50 mM Tris-HCl, 120 mM NaCl, 5 mM KCl, 2 μg/mL pepstatin A, with Complete Mini™ protease inhibitor and PhosSTOP™ phosphatase inhibitor tablets from Roche Diagnostics, USA added at recommended dilutions), then aliquoted and stored at −75 °C until use. To obtain insoluble PHF fractions and soluble cytosolic fractions, dissected brain pieces were dispersed in a glass tissue grinder at 50 mg tissue wet weight/mL in ice-cold mild extraction buffer (250 mM sucrose, 20 mM HEPES pH 7.4, 10 mM KCl, 1.5 mM MgCl_2_, 1 mM EDTA, 1 mM EGTA, 1 mM DTT, with Complete Mini™ protease inhibitor and PhosSTOP™ phosphatase inhibitor tablets from Roche Diagnostics, USA added at recommended dilutions). Dispersed lysates were passed through a 26G needle 10 times using 1 mL syringes. Lysates were first centrifuged at 800 × g for 5 min at 4 °C, then at 10,000 × g for 10 min at 4 °C, with the resulting supernatant designated as the cytosolic soluble fraction (after further centrifugation at 100,000 × g for 1 h at 4 °C) and stored at −75 °C until use. Pellets resulting from the 800 × g centrifugation step (see above) were resuspended in 1 % sarkosyl Tris-buffer, centrifuged (160,000 × g, 50 min 4 °C) with further suspension in 5 M guanidine buffer for 3 h at 25 °C, then centrifuged again (100,000 × g, 30 min 4 °C) before the final supernatant was designated as the insoluble sarkosyl PHD fraction [55] and precipitated using ethanol before immunoblotting. Protein concentrations were measured using Coomassie Plus™ Assay kits (Thermo Scientific, USA). For immunoblotting, total or fractionated lysates were mixed 1:1 with Laemmli Sample Buffer (Bio-Rad, USA) and heated to 95 °C for 5mins. For ELISA assays, homogenates were processed in ice-cold 5 M guanidine HCl/50 mM Tris–HCl, pH 8.0 as previously described [54].

### Immunoblotting

Samples were loaded on a 10 or 12 % SDS-polyacrylamide gel and transferred to nitrocellulose membrane using the iBlot® dry-transfer system (Invitrogen, USA). Membranes were blocked with 5 % bovine serum albumin (BSA) in 20 mM Tris-buffered saline, pH 7.6 with 0.1 % Tween® 20 (TBST) at 25 °C for 1 h, then incubated with primary antibodies overnight at 4 °C degree in 5 % BSA in TBST at specified dilutions (see Table 2). At the end of primary antibody incubation, membranes were washed with TBST for 4 × 10 min and incubated with the appropriate horseradish peroxidase (HRP)-conjugated secondary antibodies (1: 5,000 dilution, Jackson ImmunoResearch, USA) for 1 h at 25 °C. Immunoreactivities of antibodies listed in Table 2, including GAPDH used as loading control, were visualized with Luminata™ Forte or Crescendo Western HRP substrate (Merck Millipore, Germany) and quantified with the Alliance 4.7 image analyser (UVItec, UK).

**Table 2:** Primary antibodies used for immunoblotting in this study

Antibody (catalogue no.)	Species	Dilution	Company
β-Actin (#2228)	mouse mAb	1:5000	Sigma
CDK5 (#2506)	rabbit pAb	1:1000	CST
CRMP2, clone C4G (#11096)	mouse mAb	1:1000	IBL
CRMP2, pSer522 (#CP2191)	rabbit pAb	1:1000	ECM
CRMP2, pThr514 (#9397)	rabbit pAb	1:1000	CST
CRMP2, phosphorylated clone 3 F4 (#29060)	mouse mAb	1:200	IBL
GAPDH (#G8795)	mouse mAb	1:10000	Sigma
GSK3β, clone 3D10 (#9832)	mouse mAb	1:500	CST
GSK3β, pSer9 (#5558)	rabbit mAb	1:2000	CST
GSK3β, pTyr216 (#ab75745)	rabbit pAb	1:2000	Abcam
p35 (#sc-820)^a^	rabbit pAb	1:200	SC
PP2A, C subunit (#2038)	rabbit pAb	1:1000	CST
Synaptophysin (#MAB368)	mouse mAb	1:2500	MM
α-Synuclein (#sc-7011-R)	rabbit pAb	1:500	SC
β-III-Tubulin, clone TU-20 (#ab7751)	mouse mAb	1:1000	Abcam

*mAB* monoclonal antibodies, *pAB* polyclonal antibodies. Company abbreviations: *Abcam* Abcam plc (UK), *CST* Cell Signaling Technology (USA), *ECM* ECM Biosciences (USA), *IBL* IBL-International (Germany), *MM* Merck-Millipore (Germany), *SC* Santa Cruz Biotechnology (USA), *Sigma* Sigma-Aldrich (USA)

^a^Also recognizes p25

### ELISA assays

Aβ_40_ and Aβ_42_ concentrations were assessed in duplicates in aliquots of cytosolic soluble fractions using ELISA kits (KHB3482 and KHB3442, ThermoFisher Scientific, USA) according to manufacturer’s instructions. Ratios of Aβ_42_ to Aβ_40_ were calculated by dividing values of Aβ_42_ by Aβ_40_. Similarly, pSer396 tau and total tau were assayed using ELISA kits (KHB7031 and KHB0041, ThermoFisher Scientific, USA), and ratios of pSer396 tau to total tau were calculated.

### Data analysis

Data was analyzed using SPSS 20.0 (IBM, USA) or Prism 6.0 (GraphPad, USA) software. Comparisons between groups were assessed with parametric (one way analyses of variance, ANOVA with Bonferroni *post*-*hoc* corrections) or non-parametric (Kruskal-Wallis H tests with Dunn’s *post*-*hoc* corrections) based on normality of the data, as assessed by Kolmogorov-Smirnov tests. Due to the ordinal nature of some of the variables, all correlations were based on non-parametric Spearman rank coefficients. A *p*-value < 0.05 is considered statistically significant. As the study is exploratory in nature, no adjustment for multiple correlations were made.

## Additional files

Additional file 1: Figure S1. CRMP2 is concentrated in cytosol-enriched fractions of postmortem human neocortex. Representative CRMP2 immunoblots of total and cytosol-enriched (“Cytosolic”) fractions of postmortem human neocortex (5 μg protein loaded per lane). β-actin was used as a control to show concentration of soluble cytoskeletal proteins, including CRMP2, in the cytosol-enriched fractions using the fractionation procedure described in Methods. (PDF 116 kb) Additional file 2: Figure S2. pThr514 CRMP2 is increased in total homogenate fractions of DLB. a Representative immunoblots (with molecular weight indicators in kDa to the left) and b bar graphs of immunoreactivities of total CRMP2 and pThr514 CRMP2 normalized to total CRMP2 (mean ± SEM in arbitrary units) in total brain homogenate fractions, with GAPDH used as loading control. Available *N* for control (C) = 19; PDD (P) = 19 and DLB (D) = 20. ***p* < 0.01, significant difference for multiple pair-wise comparisons (one-way ANOVA with Bonferroni *post*-*hoc* tests). (PDF 145 kb) Additional file 3: Figure S3. No correlation of pSer522, pThr509 and 3 F4 CRMP2 immunoreactivities with Aβ_42_ : Aβ_40_ in LBD parietal cortex. Scatter plots of soluble Aβ_42_ to Aβ_40_ ratio and pCRMP2 phosphorylation at a Ser522, b Thr509 and c 3 F4 within the combined LBD (DLB + PDD), DLB and PDD groups. Correlations were assessed by Spearman correlation, with insets indicating *rho* and *p* values. No significant correlation was observed. (PDF 115 kb) Additional file 4: Figure S4. No correlation between pThr514 CRMP2 and pSer396 tau in LBD parietal cortex. a Bar graph of mean (± SEM) pSer396 tau to total tau ratios. Scatter plots of pThr514 CRMP2 with pSer396 tau : total tau in total homogenate fractions of b LBD (DLB + PDD), c DLB and d PDD parietal cortex, with insets indicating *rho* and *p* values. Available *N* for control = 19; PDD = 19 and DLB = 20. No significant differences (*p* > 0.05) were found for multiple pair-wise comparisons of pSer396 : total tau between groups (Kruskal-Wallis H tests), or for pThr514 CRMP2 correlations with pSer396 tau : total tau ratios (Spearman). (PDF 106 kb) Additional file 5: Figure S5. No correlation between pThr514 CRMP2 and α-synuclein immunoreactivity in LBD parietal cortex. a Bar graph of α-synuclein immunoreactivity (mean ± SEM in arbitrary units) with representative immunoblots, with GAPDH as loading control. Scatter plots of pThr514 CRMP2 with α-synuclein immunoreactivity in total homogenate fractions of b LBD (DLB + PDD), c DLB and d PDD parietal cortex, with insets indicating *rho* and *p* values. Available *N* for control (C) = 19; PDD (P) = 19 and DLB (D) = 20. No significant differences (*p* > 0.05) were found for multiple pair-wise comparisons of α-synuclein between groups (one-way ANOVA with Bonferroni’s *post*-*hoc* tests), or for pThr514 CRMP2 correlations with α-synuclein (Spearman). (PDF 195 kb) Additional file 6: Figure S6. No correlation between pThr514 CRMP2 and insoluble pSer129 α-synuclein immunoreactivity in LBD parietal cortex. a Bar graphs of pSer129 α-synuclein normalized to α-synuclein immunoreactivities in the insoluble fraction (mean ± SEM in arbitrary units) with representative immunoblots. Scatter plots of pThr514 CRMP2 with pSer129 α-synuclein immunoreactivity of b LBD (DLB + PDD), c DLB and d PDD parietal cortex, with insets indicating *rho* and *p* values. Available *N* for control (C) = 19; PDD (P) = 19 and DLB (D) = 19. One DLB sample was excluded as an outlier due to pSer129 α-synuclein value > 8, the inclusion of which did not alter the significance of the results (data not shown). **p* < 0.05, ***p* < 0.01, significant differences for multiple pair-wise comparisons (Kruskal-Wallis H with Dunn’s *post*-*hoc* tests). No significant differences (*p* > 0.05) were found for pThr514 CRMP2 correlations with pSer129 α-synuclein (Spearman). (PDF 86 kb) Additional file 7: Figure S7. Unchanged PP2A C-subunit immunoreactivity in DLB parietal cortex. a Representative immunoblots and b bar graphs of PP2A C-subunit immunoreactivity (mean ± SEM in arbitrary units), with GAPDH as loading control. Available *N* for control (C) = 19; PDD (P) = 19 and DLB (D) = 20. No significant differences (*p* > 0.05) were found for multiple pair-wise comparisons of PP2A C-subunit between groups (one-way ANOVA with Bonferroni’s *post*-*doc* tests). (PDF 133 kb)

## Abbreviations

Aβ: β-amyloid
AD: Alzheimer’s disease
ANOVA: One-way analysis of variance
BSA: Bovine serum albumin
CDK5: Cyclin-dependent kinase 5
CRMP: Collapsing response mediator protein
DLB: Dementia with Lewy bodies
DTT: Dithiothreitol
EDTA: Ethylenediaminetetraacetic acid
EGTA: Triethylene glycol diaminetetraacetic acid
ELISA: Enzyme-linked immunosorbent assay
GAPDH: Glyceraldehyde 3-phosphate dehydrogenase
GSK3β: Glycogen synthase kinase 3β
HEPES: 2-[4-(2-hydroxyethyl)piperazin-1-yl]ethanesulfonic acid
HRP: Horseradish peroxidase
LBD: Lewy body dementias
PDD: Parkinson’s disease dementia
PP2A: Protein phosphatase 2A
Tris: tris(hydroxymethyl)aminomethane
