# Supplementary material for: Increased phosphorylation of collapsin response mediator protein-2 at Thr514 correlates with β-amyloid burden and synaptic deficits in Lewy body dementias
Source: Mol Brain. 2016 Sep 8;9(1):84. doi: 10.1186/s13041-016-0264-9 (PMC5016931; doi:10.1186/s13041-016-0264-9)
Supplement: Additional file 1: Figure S1. — CRMP2 is concentrated in cytosol-enriched fractions of postmortem human neocortex. Representative CRMP2 immunoblots of total and cytosol-enriched (“Cytosolic”) fractions of postmortem human neocortex (5 μg protein loaded per lane). β-actin was used as a control to show concentration of soluble cytoskeletal proteins, including CRMP2, in the cytosol-enriched fractions using the fractionation procedure described in Methods. (PDF 116 kb) [file 13041_2016_264_MOESM1_ESM.pdf]

Xing *et al.* Increased phosphorylation of collapsin response mediator protein-2 at Thr514 correlates with β-amyloid burden and synaptic deficits in Lewy Body dementias

Additional File 1: Supplementary Figure 1
CRMP2 is concentrated in cytosol-enriched fractions of postmortem human neocortex

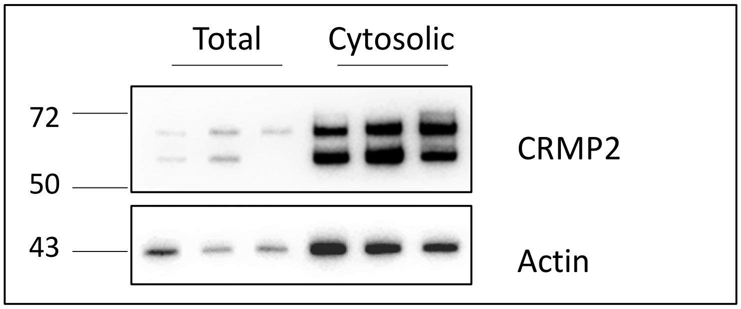

Fig. S1 Representative CRMP2 immunoblots of total and cytosol-enriched ("Cytosolic") fractions of postmortem human neocortex (5 μg protein loaded per lane). β-actin was used as a control to show concentration of soluble cytoskeletal proteins, including CRMP2, in the cytosol-enriched fractions using the fractionation procedure described in Methods.
