# Supplementary material for: Increased phosphorylation of collapsin response mediator protein-2 at Thr514 correlates with β-amyloid burden and synaptic deficits in Lewy body dementias
Source: Mol Brain. 2016 Sep 8;9(1):84. doi: 10.1186/s13041-016-0264-9 (PMC5016931; doi:10.1186/s13041-016-0264-9)
Supplement: Additional file 2: Figure S2. — pThr514 CRMP2 is increased in total homogenate fractions of DLB. a Representative immunoblots (with molecular weight indicators in kDa to the left) and b bar graphs of immunoreactivities of total CRMP2 and pThr514 CRMP2 normalized to total CRMP2 (mean ± SEM in arbitrary units) in total brain homogenate fractions, with GAPDH used as loading control. Available N for control (C) = 19; PDD (P) = 19 and DLB (D) = 20. **p < 0.01, significant difference for multiple pair-wise comparisons (one-way ANOVA with Bonferroni post-hoc tests). (PDF 145 kb) [file 13041_2016_264_MOESM2_ESM.pdf]

Xing *et al.* Increased phosphorylation of collapsin response mediator protein-2 at Thr514 correlates with β-amyloid burden and synaptic deficits in Lewy Body dementias

Additional File 2: Supplementary Figure 2
pThr514 CRMP2 is increased in total homogenate fractions of DLB

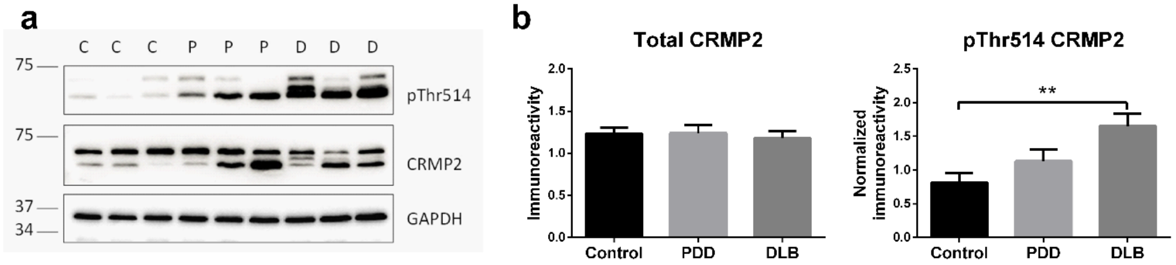

Fig. S2 a Representative immunoblots (with molecular weight indicators in kDa to the left) and **b** bar graphs of immunoreactivities of total CRMP2 and pThr514 CRMP2 normalized to total CRMP2 (mean ± SEM in arbitrary units) in total brain homogenate fractions, with GAPDH used as loading control. Available *N* for control (C) = 19; PDD (P) = 19 and DLB (D) = 20.

**p<0.01, significant difference for multiple pair-wise comparisons (one-way ANOVA with Bonferroni *post-hoc* tests).
