# Supplementary material for: Increased phosphorylation of collapsin response mediator protein-2 at Thr514 correlates with β-amyloid burden and synaptic deficits in Lewy body dementias
Source: Mol Brain. 2016 Sep 8;9(1):84. doi: 10.1186/s13041-016-0264-9 (PMC5016931; doi:10.1186/s13041-016-0264-9)
Supplement: Additional file 3: Figure S3. — No correlation of pSer522, pThr509 and 3 F4 CRMP2 immunoreactivities with Aβ42 : Aβ40 in LBD parietal cortex. Scatter plots of soluble Aβ42 to Aβ40 ratio and pCRMP2 phosphorylation at a Ser522, b Thr509 and c 3 F4 within the combined LBD (DLB + PDD), DLB and PDD groups. Correlations were assessed by Spearman correlation, with insets indicating rho and p values. No significant correlation was observed. (PDF 115 kb) [file 13041_2016_264_MOESM3_ESM.pdf]

Xing *et al.* Increased phosphorylation of collapsin response mediator protein-2 at Thr514 correlates with β-amyloid burden and synaptic deficits in Lewy Body dementias

Additional File 3: Supplementary Figure 3

No correlation of pSer522, pThr509 and 3F4 CRMP2 immunoreactivities with $A\beta_{42}$: $A\beta_{40}$ in LBD parietal cortex

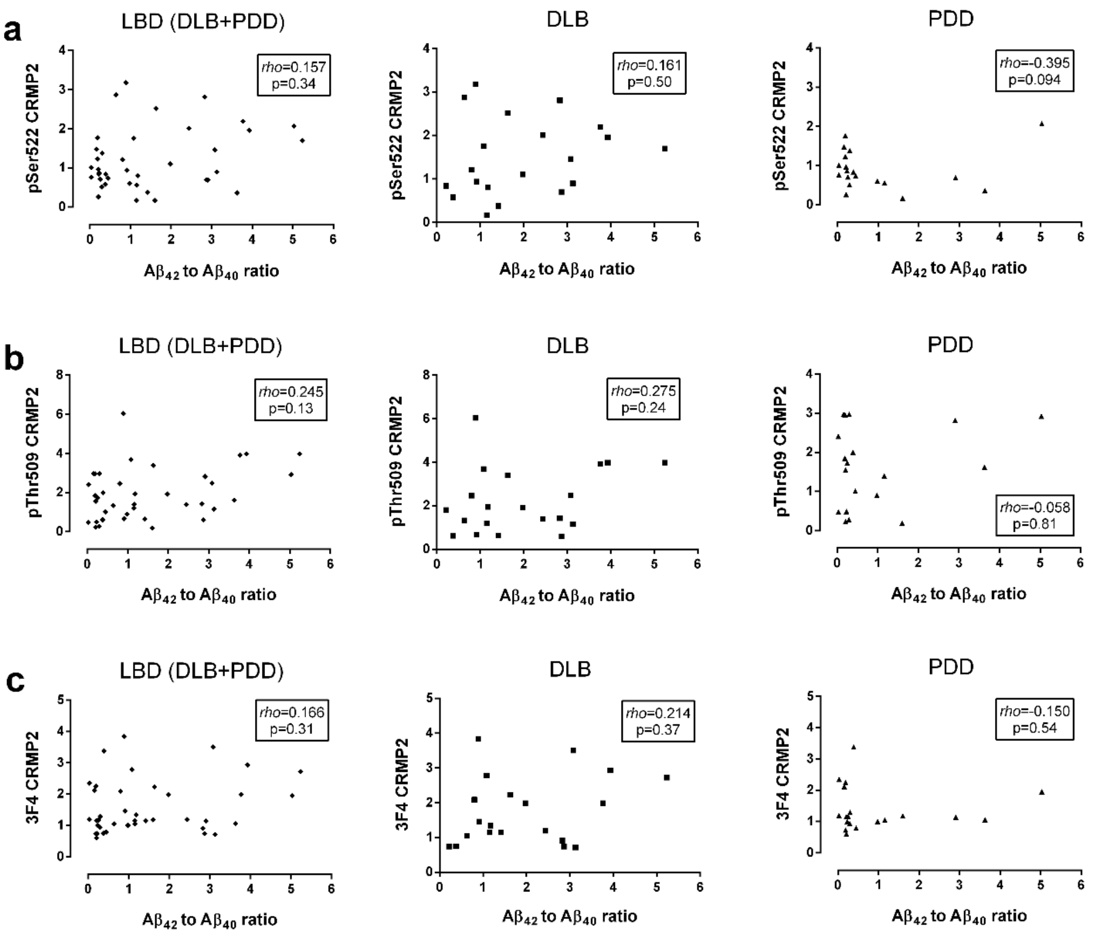

Fig. S3 Scatter plots of soluble $A\beta_{42}$ to $A\beta_{40}$ ratio and pCRMP2 phosphorylation at **a** Ser522, **b** Thr509 and **c** 3F4 within the combined LBD (DLB+PDD), DLB and PDD groups. Correlations were assessed by Spearman correlation, with insets indicating *rho* and p values. No significant correlation was observed.
