# Supplementary material for: Increased phosphorylation of collapsin response mediator protein-2 at Thr514 correlates with β-amyloid burden and synaptic deficits in Lewy body dementias
Source: Mol Brain. 2016 Sep 8;9(1):84. doi: 10.1186/s13041-016-0264-9 (PMC5016931; doi:10.1186/s13041-016-0264-9)
Supplement: Additional file 4: Figure S4. — No correlation between pThr514 CRMP2 and pSer396 tau in LBD parietal cortex. a Bar graph of mean (± SEM) pSer396 tau to total tau ratios. Scatter plots of pThr514 CRMP2 with pSer396 tau : total tau in total homogenate fractions of b LBD (DLB + PDD), c DLB and d PDD parietal cortex, with insets indicating rho and p values. Available N for control = 19; PDD = 19 and DLB = 20. No significant differences (p > 0.05) were found for multiple pair-wise comparisons of pSer396 : total tau between groups (Kruskal-Wallis H tests), or for pThr514 CRMP2 correlations with pSer396 tau : total tau ratios (Spearman). (PDF 106 kb) [file 13041_2016_264_MOESM4_ESM.pdf]

Xing *et al.* Increased phosphorylation of collapsin response mediator protein-2 at Thr514 correlates with β-amyloid burden and synaptic deficits in Lewy Body dementias

Additional File 4: Supplementary Figure 4
No correlation between pThr514 CRMP2 and pSer396 tau in LBD parietal cortex

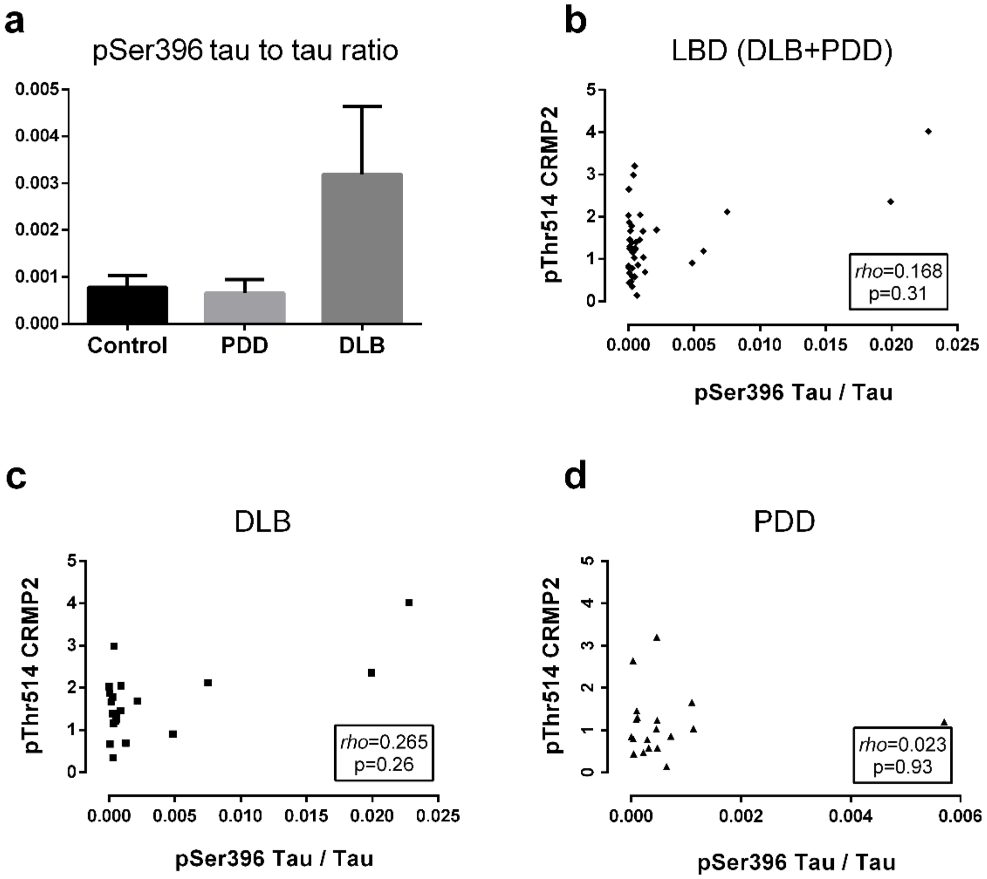

Fig. S4 a Bar graph of mean (± SEM) pSer396 tau to total tau ratios. Scatter plots of pThr514 CRMP2 with pSer396 tau : total tau in total homogenate fractions of **b** LBD (DLB + PDD), **c** DLB and **d** PDD parietal cortex, with insets indicating *rho* and p values. Available *N* for control = 19; PDD = 19 and DLB = 20. No significant differences ($p>0.05$) were found for multiple pair-wise comparisons of pSer396 : total tau between groups (Kruskal-Wallis H tests), or for pThr514 CRMP2 correlations with pSer396 tau : total tau ratios (Spearman).
