# Supplementary material for: Increased phosphorylation of collapsin response mediator protein-2 at Thr514 correlates with β-amyloid burden and synaptic deficits in Lewy body dementias
Source: Mol Brain. 2016 Sep 8;9(1):84. doi: 10.1186/s13041-016-0264-9 (PMC5016931; doi:10.1186/s13041-016-0264-9)
Supplement: Additional file 5: Figure S5. — No correlation between pThr514 CRMP2 and α-synuclein immunoreactivity in LBD parietal cortex. a Bar graph of α-synuclein immunoreactivity (mean ± SEM in arbitrary units) with representative immunoblots, with GAPDH as loading control. Scatter plots of pThr514 CRMP2 with α-synuclein immunoreactivity in total homogenate fractions of b LBD (DLB + PDD), c DLB and d PDD parietal cortex, with insets indicating rho and p values. Available N for control (C) = 19; PDD (P) = 19 and DLB (D) = 20. No significant differences (p > 0.05) were found for multiple pair-wise comparisons of α-synuclein between groups (one-way ANOVA with Bonferroni’s post-hoc tests), or for pThr514 CRMP2 correlations with α-synuclein (Spearman). (PDF 195 kb) [file 13041_2016_264_MOESM5_ESM.pdf]

Xing *et al.* Increased phosphorylation of collapsin response mediator protein-2 at Thr514 correlates with β-amyloid burden and synaptic deficits in Lewy Body dementias

Additional File 5: Supplementary Figure 5

No correlation between pThr514 CRMP2 and α-synuclein immunoreactivity in LBD parietal cortex

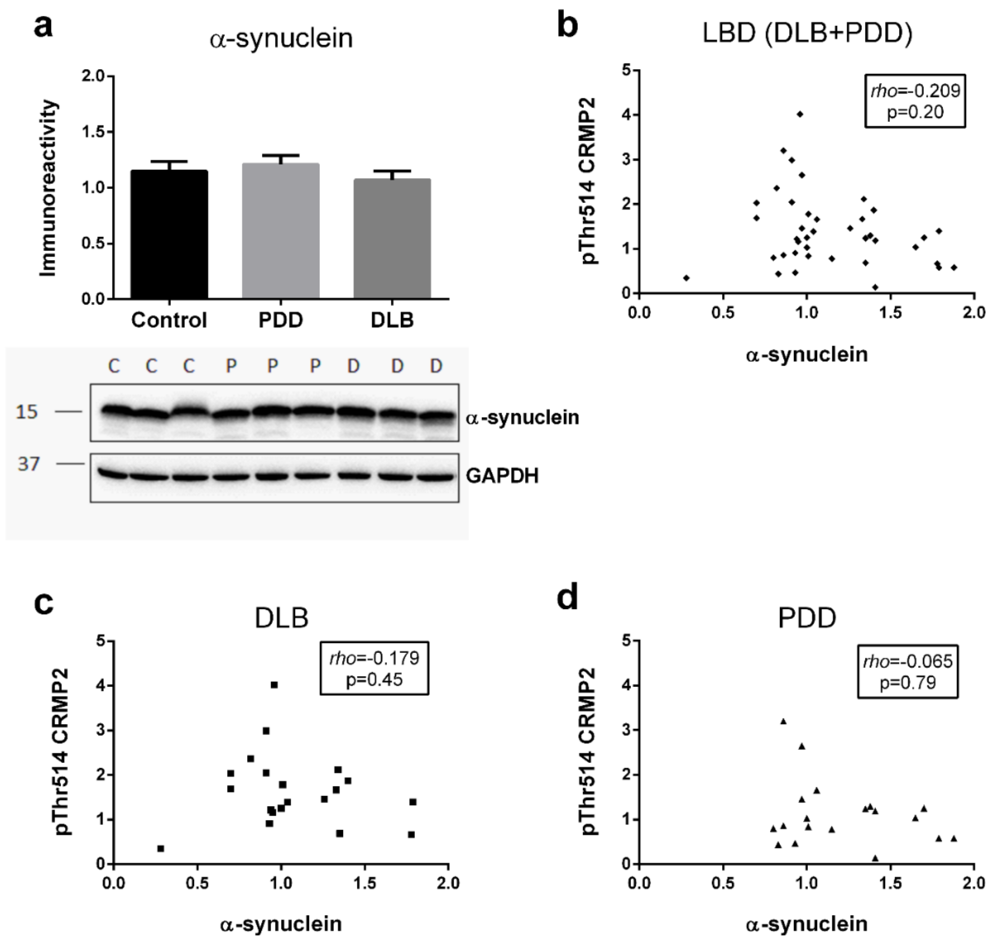

Fig. S5 a Bar graph of α-synuclein immunoreactivity (mean ± SEM in arbitrary units) with representative immunoblots, with GAPDH as loading control. Scatter plots of pThr514 CRMP2 with α-synuclein immunoreactivity in total homogenate fractions of **b** LBD (DLB + PDD), **c** DLB and **d** PDD parietal cortex, with insets indicating *rho* and p values. Available *N* for control (C) = 19; PDD (P) = 19 and DLB (D) = 20. No significant differences ($p>0.05$) were found for multiple pair-wise comparisons of α-synuclein between groups (one-way ANOVA with Bonferroni's *post-hoc* tests), or for pThr514 CRMP2 correlations with α-synuclein (Spearman).
