# Supplementary material for: Increased phosphorylation of collapsin response mediator protein-2 at Thr514 correlates with β-amyloid burden and synaptic deficits in Lewy body dementias
Source: Mol Brain. 2016 Sep 8;9(1):84. doi: 10.1186/s13041-016-0264-9 (PMC5016931; doi:10.1186/s13041-016-0264-9)
Supplement: Additional file 6: Figure S6. — No correlation between pThr514 CRMP2 and insoluble pSer129 α-synuclein immunoreactivity in LBD parietal cortex. a Bar graphs of pSer129 α-synuclein normalized to α-synuclein immunoreactivities in the insoluble fraction (mean ± SEM in arbitrary units) with representative immunoblots. Scatter plots of pThr514 CRMP2 with pSer129 α-synuclein immunoreactivity of b LBD (DLB + PDD), c DLB and d PDD parietal cortex, with insets indicating rho and p values. Available N for control (C) = 19; PDD (P) = 19 and DLB (D) = 19. One DLB sample was excluded as an outlier due to pSer129 α-synuclein value > 8, the inclusion of which did not alter the significance of the results (data not shown). *p < 0.05, **p < 0.01, significant differences for multiple pair-wise comparisons (Kruskal-Wallis H with Dunn’s post-hoc tests). No significant differences (p > 0.05) were found for pThr514 CRMP2 correlations with pSer129 α-synuclein (Spearman). (PDF 86 kb) [file 13041_2016_264_MOESM6_ESM.pdf]

Xing *et al.* Increased phosphorylation of collapsin response mediator protein-2 at Thr514 correlates with β-amyloid burden and synaptic deficits in Lewy Body dementias

Additional File 6: Supplementary Figure 6

No correlation between pThr514 CRMP2 and insoluble pSer129 α-synuclein immunoreactivity in LBD parietal cortex

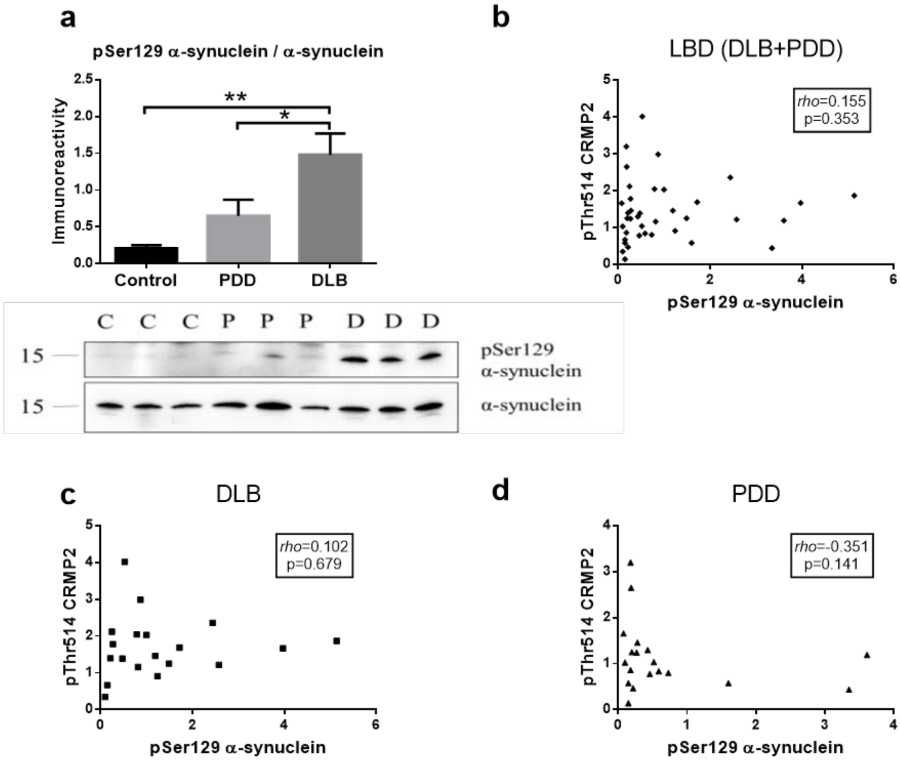

Fig. S6 a Bar graphs of pSer129 α-synuclein normalized to α-synuclein immunoreactivities in the insoluble fraction (mean ± SEM in arbitrary units) with representative immunoblots. Scatter plots of pThr514 CRMP2 with pSer129 α-synuclein immunoreactivity of **b** LBD (DLB + PDD), **c** DLB and **d** PDD parietal cortex, with insets indicating *rho* and p values. Available *N* for control (C) = 19; PDD (P) = 19 and DLB (D) = 19. One DLB sample was excluded as an outlier due to pSer129 α-synuclein value > 8, the inclusion of which did not alter the significance of the results (data not shown).

*p<0.05, **p<0.01, significant differences for multiple pair-wise comparisons (Kruskal-Wallis H with Dunn's *post-hoc* tests). No significant differences (p>0.05) were found for pThr514 CRMP2 correlations with pSer129 α-synuclein (Spearman).
