# Supplementary material for: Increased phosphorylation of collapsin response mediator protein-2 at Thr514 correlates with β-amyloid burden and synaptic deficits in Lewy body dementias
Source: Mol Brain. 2016 Sep 8;9(1):84. doi: 10.1186/s13041-016-0264-9 (PMC5016931; doi:10.1186/s13041-016-0264-9)
Supplement: Additional file 7: Figure S7. — Unchanged PP2A C-subunit immunoreactivity in DLB parietal cortex. a Representative immunoblots and b bar graphs of PP2A C-subunit immunoreactivity (mean ± SEM in arbitrary units), with GAPDH as loading control. Available N for control (C) = 19; PDD (P) = 19 and DLB (D) = 20. No significant differences (p > 0.05) were found for multiple pair-wise comparisons of PP2A C-subunit between groups (one-way ANOVA with Bonferroni’s post-doc tests). (PDF 133 kb) [file 13041_2016_264_MOESM7_ESM.pdf]

Xing *et al.* Increased phosphorylation of collapsin response mediator protein-2 at Thr514 correlates with β-amyloid burden and synaptic deficits in Lewy Body dementias

Additional File 7: Supplementary Figure 7
Unchanged PP2A C-subunit immunoreactivity in DLB parietal cortex

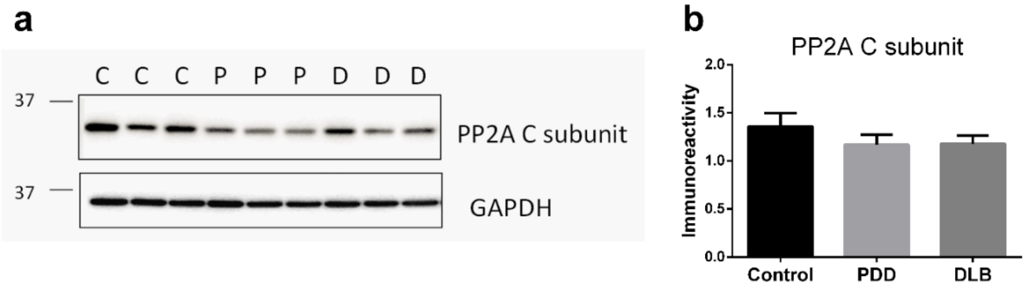

Fig. S7 a Representative immunoblots and **b** bar graphs of PP2A C-subunit immunoreactivity (mean ± SEM in arbitrary units), with GAPDH as loading control. Available *N* for control (C) = 19; PDD (P) = 19 and DLB (D) = 20. No significant differences ($p>0.05$) were found for multiple pair-wise comparisons of PP2A C-subunit between groups (one-way ANOVA with Bonferroni's *post-doc* tests).
